# A Case of Severe Pulmonary Metastasis in Advanced Endometrial Cancer and the Importance of Clinical Screening

**DOI:** 10.7759/cureus.90497

**Published:** 2025-08-19

**Authors:** Kevin T Dao, Marina Guirguis, Matthew Palmbach, Ayesha Rehman, Saakshi Dulani, Wilbur Montana, Kasey Fox, Mustafa A Alkhreisat

**Affiliations:** 1 Internal Medicine, University of California, Los Angeles (UCLA) Kern Medical, Bakersfield, USA; 2 Oncology, University of California, Los Angeles (UCLA) Kern Medical, Bakersfield, USA

**Keywords:** clinical screening, endometrial adenocarcinoma, endometrial cancer, endometrial cancer screening, health recommendations, medical prevention, metastatic lung disease, patient education

## Abstract

Endometrial cancer is one of the prevalent malignancies of the female reproductive system, with most cases presenting as low-grade tumors, especially if it is diagnosed during the early stages. As such, detection typically results in a good prognosis with the disease generally confined to the uterus. Unfortunately, recurrence and metastasis can still occur in a subset of patients who do not have proper medical follow-up or refuse to follow up, and when metastasis does occur, prognosis tends to be poor. Here, we would like to present a very severe case of endometrial cancer that has diffusely metastasized to the lungs. The purpose of this article is to emphasize the importance of medical check-ups, screening, and management in patients, especially with post-menopausal bleeding.

## Introduction

As one of the leading gynecologic malignancies worldwide, endometrial cancer continues to rise in incidence, particularly in developed countries, with obesity being one of the primary co-morbidities [1]. Fortunately, the majority of cases have an overall five-year survival range from approximately 74% to 91% in patients who do not have any metastatic disease [2]. Despite this, a proportion of patients can develop metastatic disease, especially if they do not have proper medical follow-up or evaluation. In the majority of cases, there tends to be a regional spread involving the pelvic or para-aortic lymph nodes, but distant metastases are also observed. The most frequently observed organs involved are usually the lungs, but they can also involve the liver, bones, and brain [3]. The pulmonary system, in fact, is one of the organs that has shown a high mortality rate [3]. Fortunately, however, the progression of endometrial carcinoma generally takes years to progress before advancing to stage 4, depending on whether the cancer is low or high grade. As such, patients with any abnormal uterine bleeding, especially in post-menopausal women, should have proper medical check-ups [4]. Unfortunately, endometrial cancer is not as commonly screened as cervical cancer [5,6]. In fact, despite endometrial cancer being the most common gynecologic malignancy in women in developed countries, there is unfortunately little to no standard screening protocol for the disease among asymptomatic patients in the general population in juxtaposition to cervical cancer [7], although there are methods to assist in the diagnosis particularly endometrial biopsy and/or transvaginal ultrasonography for initial evaluation [7]. Yet, for initial screening, physicians should be prudent to ask about abnormal symptoms such as post-menopausal vaginal bleeding to ensure such diseases do not progress unfettered. As such, we would like to present a severe case of a patient who came to the emergency room with initial concerns about infection or pulmonary embolism due to the acuity of her presentation. However, further workup revealed that the patient had severe metastatic lung disease with primary endometrial adenocarcinoma. A discussion regarding the importance of endometrial cancer screening and the severity of how far this disease can progress will also be discussed.

## Case presentation

The patient is a 55-year-old woman with a past medical history of diabetes mellitus and hypertension who presented to the emergency department with complaints of weakness and lightheadedness for five days, which progressed with three days of shortness of breath. She reports that initially her symptoms of weakness and dizziness occurred only when the patient was moving from a sitting to a standing position, but she reports general fatigue. She stated that over the past three days, she had experienced increasing shortness of breath, which worsened with exertion and was associated with occasional right-sided chest tightness as well as discomfort in deep breathing. Fortunately, she states that she is able to still walk around to do her activities in daily living, but her energy has been significantly inhibited by feelings of fatigue. The patient noted abnormal vaginal bleeding as well as post-coital bleeding for the past year. She otherwise denies any fever, chills, diarrhea, abdominal pain, dysuria, hematuria, or any other symptoms at this current time and notes that she does not follow up regularly with her primary care physician.

Her vitals show that she was mildly hypertensive with systolic blood pressure in the 140s and diastolic blood pressure in the 70s to 80s, as well as tachycardia with a heart rate in the low 100s, along with mild tachypnea with a respiratory rate in the low 20s. The patient was saturating with a peripheral oxygen saturation in the low to mid 90s, requiring 3 L of nasal cannula. Her body mass index was 33.9. The pulmonary exam revealed that the patient had decreased breath sounds at the bases of the lungs, both anteriorly and posteriorly. The rest of the physical exam was unremarkable.

The basic metabolic panel depicted hypercalcemia, elevated alkaline phosphatase, and elevated lactic acid with unremarkable troponins. The urinary toxicology screen was positive for amphetamines and opiates. The complete blood count had an elevated white blood cell count of 25,000 with no bandemia. Her BioFire was noted to be unremarkable. Tuberculosis QuantiFERON Gold as well as all other tuberculosis testing was noted to be unremarkable (Table 1). Blood cultures, urine culture, and sputum culture with Gram stain were drawn, and the patient started on levofloxacin 750 mg daily as well as azithromycin 500 mg daily for concerns of community-acquired pneumonia.

**Table 1:** Initial blood work

Parameter	Value	Reference
Sodium	135 mmol/L	136–145 mmol/L
Potassium	4.8 mmol/L	3.5–5.1 mmol/L
Chloride	104 mmol/L	98–107 mmol/L
Calcium (corrected)	12.9 mg/dL	8.5–10.1 mg/dL
Magnesium	2.4 mg/dL	1.8–2.4 mg/dL
Phosphorus	0.9 mg/dL	2.5–4.9 mg/dL
Alanine transaminase	19 U/L	13–61 U/L
Aspartate transaminase	32 U/L	15-37 U/L
Total bilirubin	1.3 mg/dL	0-1 mg/dL
Albumin	2.1 g/dL	3.4–5 g/dL
White blood count (WBC)	25 × 10^3^/mcL	4.5-11 × 10^3^/mcL
Hemoglobin (Hgb)	12.8 × 10^3^/mcL	13.2–17.4 g/dL
Mean corpuscular value (MCV)	88.1 HI	80–98 HI
Platelet	335 × 10^3^/mcL	150–450 × 10^3^/mcL
Neutrophil %	87.2%	50%–75%
Band %	2%	<12%
Lymphocyte %	6.0%	20%–45%
Monocyte %	6.2%	2%-12%
Eosinophil %	0.3%	<6%
Absolute neutrophil	21.8 × 10^3^/mcL	1.8–7.7 × 10^3^/mcL
Absolute lymphocyte	1.5 × 10^3^/mcL	1.2–4.5 × 10^3^/mcL
Absolute monocyte	1.5 × 10^3^/mcL	0.1–1 × 10^3^/mcL
Absolute eosinophil	0.1 × 10^3^/mcL	<0.7 × 10^3^/mcL
Metamyelocytes manual absolute	0.3 × 10^3^/mcL	0 × 10^3^/mcL
Monocytes manual absolute	0.3 × 10^3^/mcL	0 × 10^3^/mcL
Metamyelocytes manual %	1%	0%
Monocytes manual %	1%	0%
Large platelets	Occasional	N/A
Polychromasia	+1	N/A
Elliptocytosis	Occasional	N/A
Lactic acid	3.8 mmol/L	0.4-2.0 mmol
Parathyroid	14 pg/mL	18-88 pg/mL
Parathyroid-related protein	79 pg/mL	11-20 pg/mL
Hemoglobin A1c	9.1%	4.2%-6.3%
Amphetamine	Positive	Negative
Barbiturates	Negative	Negative
Benzodiazepine	Negative	Negative
Cocaine	Negative	Negative
Opiate	Positive	Negative
Phencyclidine (PCP)	Negative	Negative
Cannabinoid	Negative	Negative
Fentanyl	Negative	Negative
QuantiFERON Gold	Negative	Negative
Nil	0.01 IU/mL	N/A
Mitogen–nil	4.08 IU/mL	N/A
TB-nil	0 IU/mL	N/A
TB2-nil	0 IU/mL	N/A

**Figure 1:**
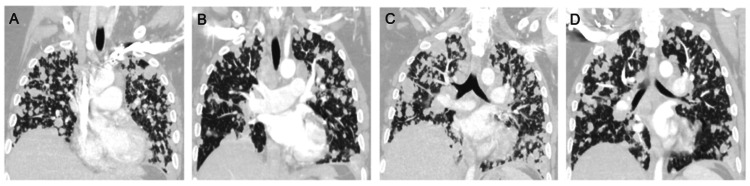
(A-D) Computer tomography angiogram of the chest with contrast No pulmonary embolism is noted, but innumerable bilateral pulmonary nodules are seen, compatible with metastatic disease

**Figure 2:**
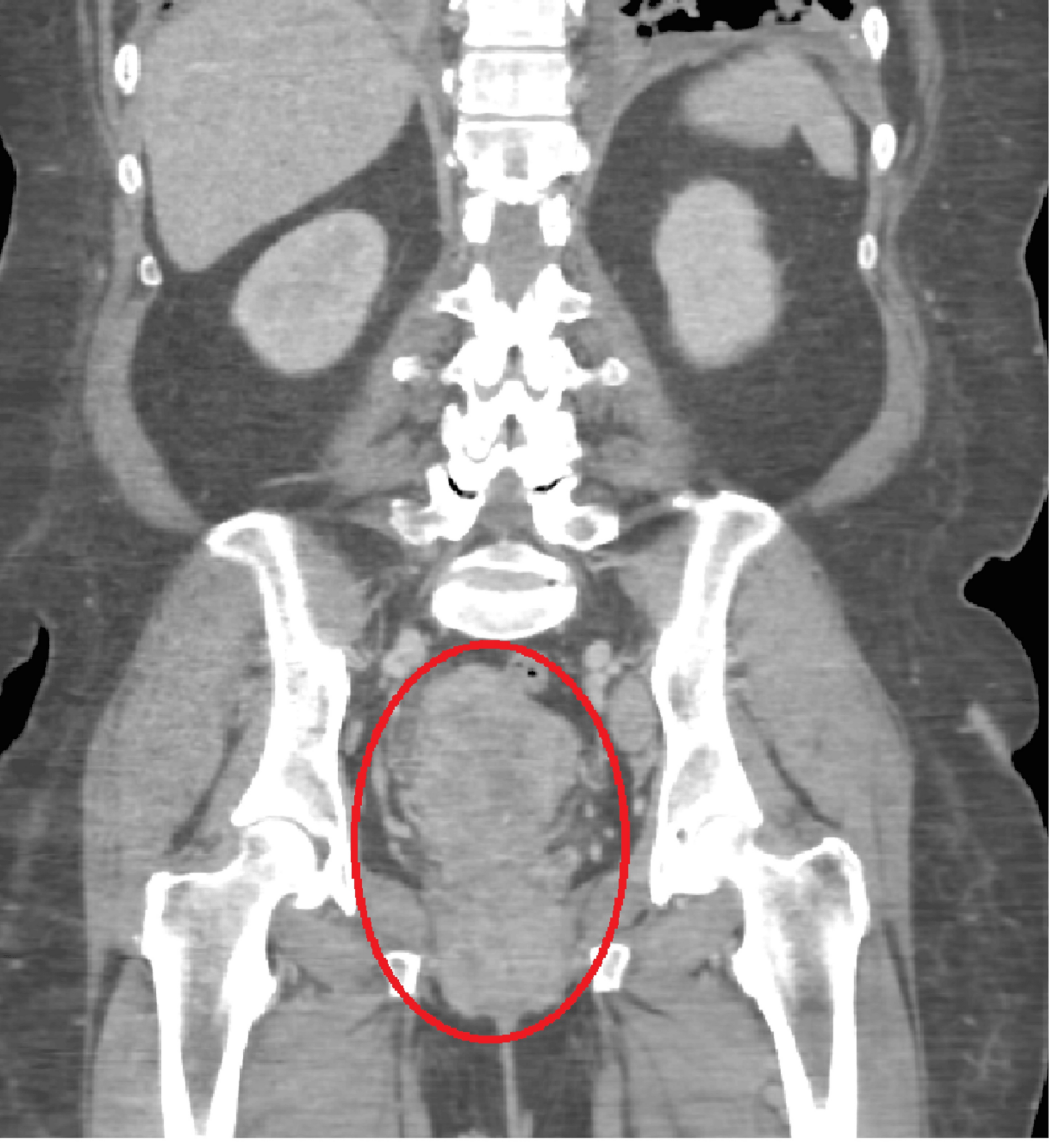
Computer tomography of the abdomen and pelvis with contrast The red circle highlights a heterogeneous appearance of the endometrium with cervical enlargement

A computer tomography angiogram of the chest with contrast was done, which showed severe diffuse pulmonary metastatic disease (Figure 1). Due to the patient also noting a history of abnormal vaginal bleeding, a computer tomography of the abdomen and pelvis with contrast was done, which showed a heterogeneous appearance of the endometrium with cervical enlargement (Figure 2). Hematology and oncology were consulted and requested more blood work along with tumor markers, particularly alpha fetal protein, CA 125, CA 19-9, carcinoembryonic antigen (CEA), beta-hCG (human chorionic gonadotropin), inhibin A/B, lactate dehydrogenase, and alkaline phosphatase along with isoenzymes (Table 2). Hematology and oncology also recommended computer tomography-guided biopsies of the endometrium and lung to ensure the patient did not have two separate primary malignancies.

**Table 2:** Further blood work ALP: alkaline phosphatase; hCG: human chorionic gonadotropin

Parameter	Value	Reference
ALP total	306 U/L	37-153 U/L
ALP intestinal isoenzyme	0%	1%-24%
ALP bone isoenzyme	36%	28%-66%
ALP liver isoenzyme	64%	25%-69%
ALP placental isoenzyme	0%	0%
ALP macrohepatic isoenzyme	Negative	Negative
ALP isoenzyme	Negative	Negative
CA 125	317 U/mL	<35 U/mL
CA 19-9	176 U/mL	<34 U/mL
Inhibin A	1 pg/mL	N/A
Inhibin B	21 pg/mL	N/A
Beta-hCG	<1	0-5

**Figure 3:**
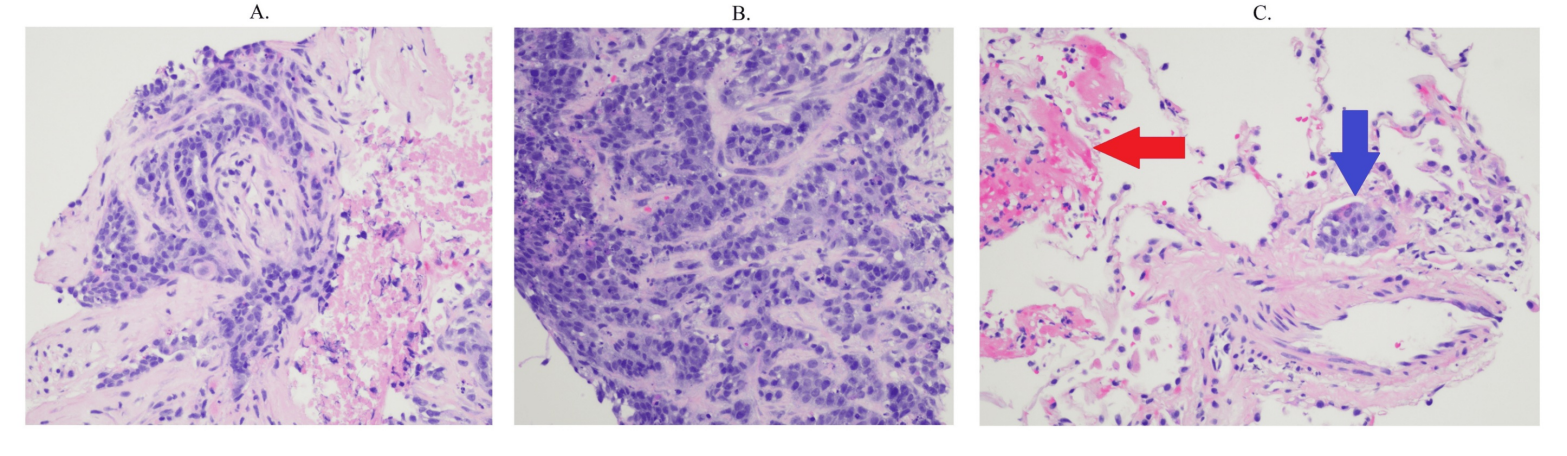
Lung nodule biopsy (A, B) Biopsy of the right upper lobe of the lung shows glandular tissues throughout the entire picture. Biopsy results are consistent with metastatic adenocarcinoma consistent with an endometrioid adenocarcinoma (C) The red arrow indicates normal lung tissues, whereas the blue arrow indicates glandular tissue of the patient’s endometrioid adenocarcinoma

**Figure 4:**
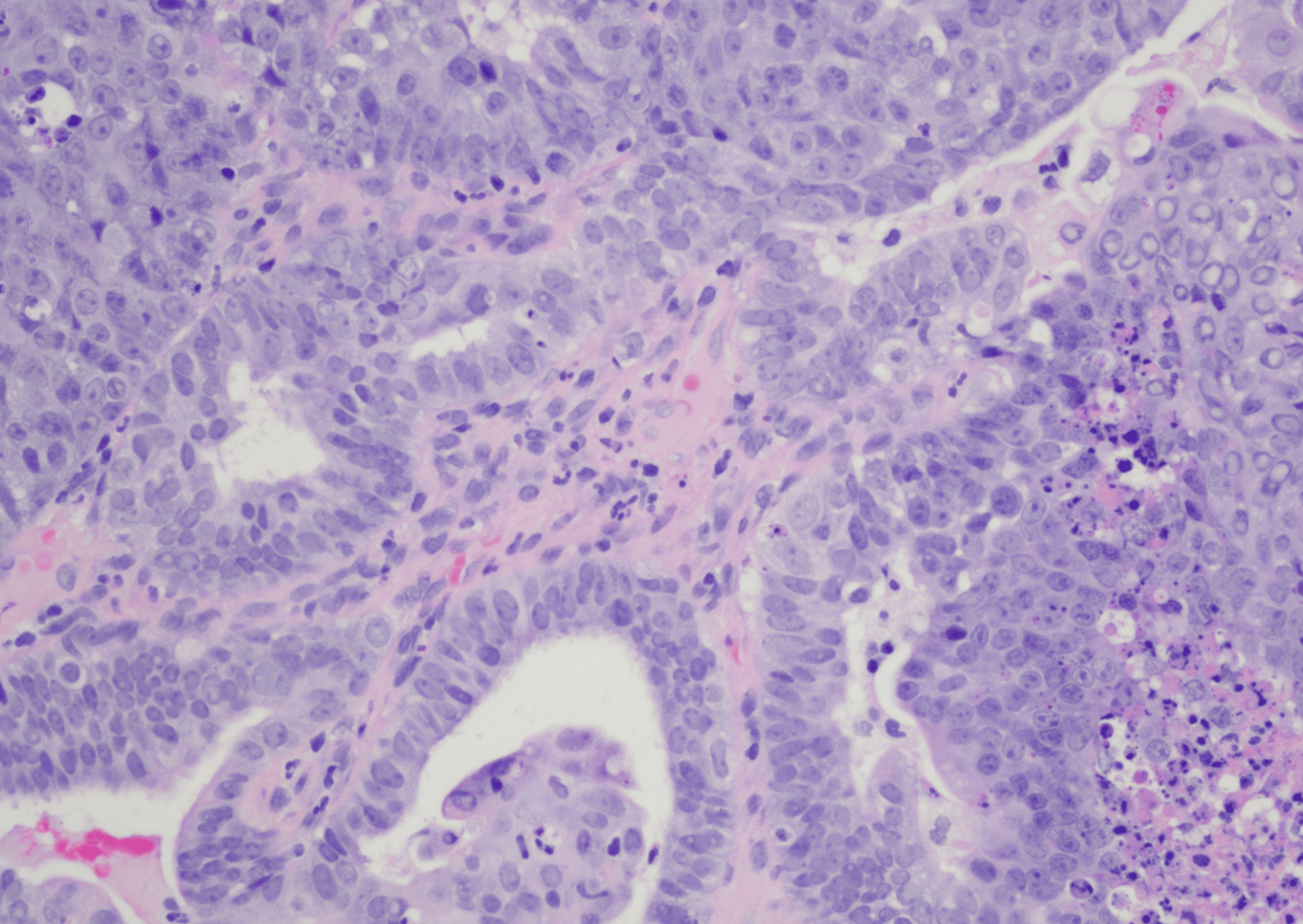
Endometrial biopsy Uterine mass biopsy was done, which indicates endometrioid adenocarcinoma, International Federation of Gynecology and Obstetrics (FIGO) grade 1

Lung nodule biopsy was obtained by interventional radiology (Figures 3A-3C) and endometrial biopsy by gynecology (Figure 4), which confirmed metastatic adenocarcinoma consistent with primary endometrioid cancer. Antibiotics were discontinued due to low suspicion of infection. Due to the patient having hypercalcemia, endocrinology was consulted, who recommended denosumab 120 mg subcutaneous one-time dose, calcitonin 4 U/kg every 12 hours for four doses, and intravenous fluids.

Unfortunately, the patient was also slowly experiencing an increase in oxygen requirement, requiring up to 35 L of oxygen via high-flow nasal cannula with a fractional inspired oxygen of 45%. A Port-a-Cath was placed; however, a plan of care discussion was held with the patient, along with a palliative care consultation. The patient states she wishes to proceed with plans for chemotherapy, but, in the case of an emergency, she wishes not to be resuscitated or intubated. Shortly after, she had increased work of breathing, was noted to be more restless and disoriented, and expired shortly after.

## Discussion

Endometrial cancer is one of the most common gynecological cancers worldwide, affecting approximately 140,000 women yearly [7,8]. In fact, the overall five-year survival rate regarding this disease is approximately 80%, with the incidence slowly rising globally [8,9]. One of the most common symptoms that occur with this disease is post-menopausal bleeding, and as such, cervical cancer screening should be done first rather than endometrial cancer screening since cervical cancer is more common worldwide [10] as opposed to endometrial cancer, which is more common in Western countries [7]. Regardless, patients who do develop this disease should be prompted to receive treatment since metastasis depicts a high mortality in most instances [3]. This unique case depicts the importance of proper medical follow-up and how, even with a disease that takes many years to progress, frequent medical appointments should be done with proper screening to ensure such a cancer does not metastasize to such a severe extent. Despite the lungs being one of the most common sites of endometrial carcinoma metastasis [3], this case shows a very extreme instance and should relay the importance that physicians should have in ensuring proper guideline medical screening. In fact, one study goes into great detail about the key concepts of oncological screening by discussing overdiagnosis, benefit and harm, cost, etc. [11]. Yet, other studies have shown mixed data, with one study involving a meta-analysis study only noting that out of all the cancer screenings, the only significant screening test with a notable lifetime gain benefit was sigmoidoscopy [12]. Such a study was limited to fecal occult blood testing (FOBT), colonoscopy, sigmoidoscopy for colon cancer, computed tomography chest screening for lung cancer, prostate-specific antigen testing for prostate cancer, and mammography screening for breast cancer [12]. Despite this study, however, large organizations such as the National Comprehensive Cancer Network and US Preventive Services Task Force have presented clear recommended guidelines on the importance of cancer screening [13-16]. Nonetheless, no studies or guidelines have been made regarding endometrial cancer screening [7]. The most common symptom associated with endometrial carcinoma is post-menopausal bleeding, and with the recommended frequency of cervical cancer screening, hopefully, such disease can be detected at earlier stages [4,14].

## Conclusions

Clinical screening and medical prevention are the classic mainstays of basic health recommendations among primary care physicians to ensure that patients do not develop severe and debilitating malignancies. This crucial fact is why multiple health organizations have developed recommendations detailing the importance of standard medical check-ups and screening. This case portrays how endometrial cancer, if undetected, can progress to a severe metastatic pulmonary disease. Thus, it should be noted that physicians should ensure proper patient education so that patients themselves can bring up any concerns during any health-related appointments. Hopefully, clinicians will continue to further encourage their patients to have proper yearly check-ups so that such diseases do not progress to this state in the future.
